# Analysis of neoadjuvant therapy effect on 30-day postoperative outcomes in gallbladder cancer

**DOI:** 10.1016/j.sopen.2024.08.001

**Published:** 2024-08-22

**Authors:** Nicole Martin, Areg Grigorian, Francesca A. Kimelman, Zeljka Jutric, Stephen Stopenski, David K. Imagawa, Ron F. Wolf, Shimul Shah, Jeffry Nahmias

**Affiliations:** a University of California, Irvine, Department of Surgery, 101 The City Dr S, Orange, CA, USA; b University of Cincinnati, Department of Surgery, 231 Albert Sabin Way, Cincinnati, OH, USA

**Keywords:** Neoadjuvant therapy, Gallbladder neoplasms, Postoperative complications, Treatment outcome, Blood transfusion

## Abstract

**Background:**

The role of neoadjuvant therapy (NAT) in gallbladder cancer (GBC) is not well established. We sought to evaluate the effect of NAT on postoperative outcomes following surgical resection of GBC. We hypothesized that patients receiving NAT would have similar rates of 30-day mortality, readmission, and postoperative complications (e.g. bile leakage and liver failure) compared to those who did not receive NAT.

**Methods:**

The 2014–2017 American College of Surgeons National Surgical Quality Improvement Program (ACS-NSQIP) Procedure-Targeted Hepatectomy database was queried for patients that underwent surgery for GBC. Propensity scores were calculated to match patients in a 1:2 ratio based on age, comorbidities, functional status, and tumor staging.

**Results:**

A total of 37 patients undergoing NAT were matched to 74 patients without NAT. There was no difference in any matched characteristics. Compared to the NAT group, the no NAT cohort had similar rates of postoperative bile leakage (NAT 13.5 % vs. no NAT 10.8 %, *p* = 0.31), postoperative liver failure (5.4 %, vs. 8.1 %, *p* = 0.60), 30-day readmission (10.8 % vs. 10.8 %, *p* = 1.00), and 30-day mortality (10.8 % vs. 2.7 %, *p* = 0.075). All 30-day complications were similar except for a higher rate of postoperative blood transfusion (NAT 32.4 % vs. no NAT 10.8 %, *p* = 0.005).

**Conclusion:**

In patients undergoing surgical resection for GBC, those with and without NAT had similar rates of readmission and 30-day mortality, however NAT was associated with an increased risk for transfusion. Despite use of a large national database, this study may be underpowered to adequately assess the effect of NAT on perioperative GBC outcomes and thus warrants further investigation.

## Highlights

• Gallbladder cancer patients with vs without NAT had similar mortality, readmission, and post-operative complication rates.
• Those receiving NAT had a higher risk of requiring a post-operative blood transfusion.
• Our findings support the safety of neoadjuvant therapy in gallbladder cancer.

## Introduction

Worldwide, gallbladder cancer (GBC) is the third most common gastrointestinal tract malignancy, but the most common malignancy of the biliary tract, accounting for up to 95 % of cancers originating from the biliary system [1]. However, GBC is considered rare in the United States, with only 1.13 new cases and 0.62 deaths per 100,000 patients annually [2]. The 5-year survival rate for GBC is <5 % and mean overall survival (OS) is ~6 months for GBC patients [3]. This poor prognosis is at least partly rooted in the fact that the gallbladder's lack of a serosal layer and close contact with the liver facilitate hepatic invasion and subsequent metastatic progression [3]. The often-advanced stage and grade of GBC at the time of diagnosis contributes to its poor prognosis. In fact, 85 % of cases are either locally advanced, node positive and/or metastatic at the time of diagnosis [2]. Therefore, only 10–30 % of patients are surgical candidates based on their disease stage [[4], [5], [6]].

Neoadjuvant therapy (NAT) has become more prevalent for other gastrointestinal and hepatobiliary tumors with randomized controlled trials of NAT in esophageal, gastric, and rectal cancers showing that NAT decreases tumor size and stage and significantly improves survival rates [[7], [8], [9]]. For pancreatic cancer, Zhan et al. performed a meta-analysis of 19 prospective studies examining the effects of NAT, focusing on patients with borderline resectable and locally advanced pancreatic cancer. They found that patients who received NAT had overall resection and radical resection with negative margin rates of 40.2 % and 79.4 %, respectively [10].

In recent years, multiple retrospective case series have suggested the utility of NAT in downsizing advanced GBC tumors initially deemed unresectable [[11], [12], [13], [14], [15], [16]]. In fact, a systematic review by Hakeem et al. demonstrated that out of 474 patients that received NAT in 8 studies, 50.4 % were subsequently considered surgical resection candidates after NAT and 40.3 % underwent curative resection resulting in improved OS (18.5–50.1 months vs. 5.0–10.8 months in non-resected group), compared to those who remained ineligible [11]. However, all of these studies are without a comparator group.

Therefore, we sought to evaluate the effect of NAT on 30-day postoperative outcomes using a national database, hypothesizing that patients with GBC who receive NAT prior to surgery would have similar rates of postoperative bile leakage, postoperative liver failure, readmission, and 30-day mortality compared to a similarly matched cohort of patients with GBC who did not receive NAT prior to surgery.

## Materials and methods

A retrospective analysis of the American College of Surgeons National Surgical Quality Improvement Program (ACS-NSQIP) Procedure-Targeted Hepatectomy database was performed between January 2014 and December 2017. The study was deemed exempt from our Institutional Review Board and a waiver of the informed consent was granted given the use of a deidentified national database. The database was queried for all patients that underwent surgery for GBC. Propensity scores were constructed to match patients that received NAT before surgical resection with those who underwent surgery alone, in order to mimic randomization by reducing bias due to confounding variables that may predict treatment method. Patients coded as having received preoperative systemic chemotherapy, locoregional liver ablation, and/or portal vein embolization were included within the NAT group. Patients without those codes were included within the no NAT group. Patients with and without NAT were matched in a 1:2 ratio, respectively, based on preoperative characteristics including age, comorbidities, functional status, tumor staging, and preoperative biliary stent placement. Comorbidities were identified by the appropriate NSQIP variables and included ascites, bleeding disorder, dyspnea, congestive heart failure, chronic obstructive pulmonary disease, hypertension, smoking, diabetes, end-stage renal disease, and weight loss.

Operative characteristics and postoperative clinical outcomes were also compared between patients that received NAT before surgery and patients that did not (no NAT). Operative characteristics included operative approach (i.e. open vs. laparoscopic vs. robotic), extent of resection, intraoperative liver texture, number of concurrent partial resections, intraoperative ablation, use of Pringle maneuver during resection, biliary reconstruction, and operative time. The primary postoperative clinical outcomes compared between treatment groups included postoperative bile leakage, liver failure, 30-day readmission and 30-day mortality. Additional data points and outcomes evaluated include need for invasive intervention (defined as biliary stent placement, pus or bilirubin-rich fluid from drain, or other, but not surgical re-operation), hospital length of stay (LOS), peak postoperative international normalized ratio (INR), bilirubin, and creatinine, and 30-day complications. 30-Day complications included clostridium difficile infection, respiratory failure, renal failure, cerebrovascular accident, deep incisional surgical site infection (SSI), organ space SSI, superficial incisional SSI, myocardial infarction, pneumonia, urinary tract infection (UTI), pulmonary embolism, deep vein thrombosis, sepsis, septic shock, unplanned intubation, and blood transfusion. All preoperative and operative characteristics, as well as postoperative clinical outcomes, were identified by the appropriate NSQIP variables within the targeted hepatectomy dataset, with the exception of extent of resection, which was categorized based on Current Procedural Terminology (CPT) codes. Minor hepatectomy included partial lobe hepatectomy (47120), and major hepatectomy included extensive (47122), left (47125), and right hepatectomy (47130). All categorical variables including preoperative characteristics, operative characteristics, and clinical outcomes were coded as either present or absent.

Continuous variables were compared using Mann-Whitney *U* tests, and categorical variables were compared using chi-square tests. Continuous data were reported as medians with interquartile range, while categorical data were reported as percentages. All *p*-values were two-sided, with a statistical significance level of <0.05. Clinically significant differences in postoperative outcomes between groups (*p* < 0.05) were adjusted for using a multivariable logistic regression model. All statistical analyses were performed using IBM SPSS Statistics for Windows, version 24 (IBM Corporation, Armonk, USA).

## Results

### Preoperative and operative characteristics

A total of 15,748 cases were included in the ACS-NSQIP Procedure-Targeted Hepatectomy database from 2014 to 2017, 111 of which were classified as gallbladder cancers. A total of 37 patients that underwent NAT before surgical resection for GBC were propensity score matched to 74 patients who did not receive NAT before surgery for GBC. There was no statistically significant difference in any matched characteristics between treatment groups, including age, comorbidities, functional status, tumor staging, and preoperative biliary stent placement (*p* > 0.05) (Table 1). The most prevalent comorbid conditions in both groups were hypertension (NAT 4.1 % vs. no NAT 56.8 %, *p* = 0.79) and diabetes (16.2 % vs. 21.6 %, *p* = 0.50). The most common TNM tumor stages were T3 (NAT 43.2 % vs. no NAT 35.1 %, *p* = 0.51), N1 (37.8 % vs. 37.8 %, *p* = 0.83), and M0/Mx (67.6 % vs. 59.5 %, *p* = 0.13). The most common therapy in the treatment group of 37 people was preoperative systemic chemotherapy (89.2 %) (Table 2).

**Table 1:** Characteristics of patients undergoing surgical resection for gallbladder cancer with and without neoadjuvant therapy.

Characteristic	Neoadjuvant −	Neoadjuvant +	*p*-Value
	(n = 74)	(n = 37)	
Age, year, median (IQR)	69 (59, 73)	69 (57, 75)	0.946
Female, n (%)	58 (78.4 %)	24 (64.9 %)	0.127
ASA classification, n (%)			0.083
1	0	0	
2	25 (33.8 %)	6 (16.2 %)	
3	46 (62.2 %)	27 (73.0 %)	
4	2 (2.7 %)	4 (10.8 %)	
Functional status, n (%)			–
Independent	74 (100 %)	37 (100 %)	
Race, n (%)			0.767
White	47 (63.5 %)	22 (59.5 %)	
Black	8 (10.8 %)	7 (18.9 %)	
Asian	7 (9.5 %)	3 (8.1 %)	
Hispanic, n (%)	4 (6.2 %)	3 (8.8 %)	0.623
Comorbidities, n (%)			
Bleeding disorder	1 (1.4 %)	0	0.478
Dyspnea	5 (6.8 %)	0	0.106
Congestive heart failure	1 (1.4 %)	0	0.478
COPD	4 (5.4 %)	1 (2.7 %)	0.518
Hypertension	42 (56.8 %)	20 (54.1 %)	0.787
Smoker	2 (2.7 %)	1 (2.7 %)	1.00
Diabetes	16 (21.6 %)	6 (16.2 %)	0.501
Weight loss	2 (2.7 %)	3 (8.1 %)	0.196
Pre-operative biliary stent, n (%)			1.00
Endoscopic	12 (16.2 %)	6 (16.2 %)	
Percutaneous	2 (2.7 %)	1 (2.7 %)	
T-stage, n (%)			0.508
T0	2 (2.7 %)	3 (8.1 %)	
T1	4 (5.4 %)	1 (2.7 %)	
T2	24 (32.4 %)	10 (27.0 %)	
T3	26 (35.1 %)	16 (43.2 %)	
T4	4 (5.4 %)	0	
Tx	1 (1.4 %)	1 (2.7 %)	
N-stage, n (%)			0.830
N0	16 (21.6 %)	8 (21.6 %)	
N1	29 (37.8 %)	14 (37.8 %)	
N2	5 (6.8 %)	3 (8.1 %)	
Nx	10 (13.5 %)	6 (16.2 %)	
M-stage, n (%)			0.128
M0/Mx	44 (59.5 %)	25 (67.6 %)	
M1	1 (1.4 %)	3 (8.1 %)	

IQR = interquartile range, ASA = American Society of Anesthesiologists, COPD = chronic obstructive pulmonary disease.

**Table 2:** Neoadjuvant therapy in patients undergoing surgery for gallbladder cancer.

Neoadjuvant therapy	%
Preoperative systemic chemotherapy	89.2 %
Locoregional liver ablation	2.7 %
Portal vein embolization	2.7 %
Preoperative systemic chemotherapy and portal vein embolization	2.7 %

The most common operative approach in both groups was open resection (NAT 89.2 % vs. no NAT 78.4 %, *p* = 0.48), followed by laparoscopic (5.4 % vs. 6.8 %) and robotic operations (5.4 % vs. 4.1 %). There was a significant difference in major hepatectomy rate between treatment groups, in that patients who underwent extensive hepatectomies more often received NAT (NAT 16.2 % vs. no NAT 1.4 %, *p* = 0.002). However, patients who underwent minor hepatectomy had similar rates of NAT vs. no NAT (91 % vs. 81.1 %, *p* = 0.095). There was no significant difference in the remaining operative characteristics, including intraoperative liver texture, number of concurrent partial resections, concurrent intraoperative ablation, Pringle maneuver, biliary reconstruction, and total operative time (Table 3).

**Table 3:** Operative characteristics of patients undergoing surgical resection for gallbladder cancer with and without neoadjuvant therapy.

Characteristic	Neoadjuvant −	Neoadjuvant +	*p*-Value
	(n = 74)	(n = 37)	
Operative approach, n (%)			0.477
Laparoscopic	5 (6.8 %)	2 (5.4 %)	
Laparoscopic w/ open assist	2 (2.7 %)	0	
Laparoscopic w/ unplanned conversion to open	5 (6.8 %)	0	
Open	58 (78.4 %)	33 (89.2 %)	
Robotic	3 (4.1 %)	2 (5.4 %)	
Robotic w/ unplanned conversion to open	1 (1.4 %)	0	
Major hepatic resection			
Extensive hepatectomy	1 (1.4 %)	6 (16.2 %)	**0.002**
Right hepatectomy	5 (6.8 %)	1 (2.7 %)	0.373
Left hepatectomy	0 (0 %)	0 (0 %)	–
Minor hepatic resection			
Partial lobe hepatectomy	68 (91.9 %)	30 (81.1 %)	0.095
Intraoperative liver texture, n (%)			0.827
Cirrhotic	2 (2.7 %)	2 (5.4 %)	
Congested	1 (1.4 %)	1 (2.7 %)	
Fatty	9 (12.2 %)	3 (8.1 %)	
Normal	23 (32.4 %)	10 (27.0 %)	
Number of concurrent partial resections, median (IQR)	1 (0, 1)	1 (0, 1)	0.807
Concurrent intraoperative ablation, n (%)	3 (4.1 %)	1 (2.7 %)	0.719
Pringle maneuver during resection, n (%)	15 (20.3 %)	10 (27.0 %)	0.422
Biliary reconstruction, n (%)	14 (18.9 %)	8 (21.6 %)	0.742
Operative time (min), median (IQR)	243 (163, 358)	260 (179, 384)	0.314

IQR = interquartile range.

Bold indicates statistical significance (p<0.05).

### Primary and secondary clinical outcomes

Compared to the NAT group, the no NAT cohort had similar rates of postoperative bile leakage (13.5 % vs. 10.8 %, *p* = 0.31), liver failure (5.4 % vs. 8.1 %, *p* = 0.60), 30-day readmission (10.8 % vs. 10.8 %, *p* = 1.00), and 30-day mortality (10.8 % vs. 2.7 %, *p* = 0.075) (Table 4). There was no difference in rates of postoperative invasive intervention (excluding reoperation), total hospital LOS, peak postoperative INR or creatinine. The NAT group had a significantly increased median peak postoperative bilirubin (1.40 vs. 0.9, *p* = 0.042) compared to the no NAT group. All 30-day complications were similar except for a higher rate of postoperative blood transfusion in patients receiving NAT (32.4 % vs. 10.8 %, *p* = 0.005). After adjustment for anemia (hematocrit < 24 %), patients undergoing NAT continued to have a higher associated risk for postoperative blood transfusion (OR 3.58, 1.29–9.92, *p* = 0.014).

**Table 4:** Clinical outcomes of patients undergoing surgical resection for gallbladder cancer with and without neoadjuvant therapy.

Outcome	Neoadjuvant −	Neoadjuvant +	*p*-Value
	(n = 74)	(n = 37)	
LOS, days, median (IQR)	6 (4, 8)	7 (4, 9)	0.199
Need for invasive intervention postoperatively (excluding reoperation), n (%)	9 (12.2 %)	6 (16.2 %)	0.556
Pus from drain/aspirate	5 (6.8 %)	4 (10.8 %)	
Biliary stent	3 (4.1 %)	1 (2.7 %)	
Bilirubin-rich fluid from drain/aspirate	1 (1.4 %)	0	
Other	0	1 (2.7 %)	
Bile leakage, n (%)			0.313
Requiring percutaneous drainage	5 (6.8 %)	2 (5.4 %)	
Clinical diagnosis with drain	3 (4.1 %)	2 (5.4 %)	
Requiring reoperation	0	1 (2.7 %)	
Postoperative liver failure, n (%)	6 (8.1 %)	2 (5.4 %)	0.604
Peak postoperative INR, median (IQR)	1.22 (1.10, 1.50)	1.30 (1.17, 1.60)	1.00
Peak postoperative bilirubin, median (IQR)	0.90 (0.50, 1.30)	1.40 (0.50, 2.70)	**0.042**
Peak postoperative creatinine, median (IQR)	0.90 (0.74, 1.17)	1.11 (0.75, 1.56)	0.697
Any 30-day readmission, n (%)	8 (10.8 %)	4 (10.8 %)	1.00
30-day complication, n (%)			
*Clostridium difficile*	1 (2.1 %)	0	0.501
Respiratory failure	3 (4.1 %)	2 (5.4 %)	0.746
Renal failure	3 (4.1 %)	2 (5.4 %)	0.746
Cerebrovascular accident	0	1 (2.7 %)	0.155
Deep incisional SSI	1 (1.4 %)	0	0.478
Myocardial infarction	0	0	–
Organ space SSI	10 (13.5 %)	4 (10.8 %)	0.686
Pneumonia	2 (2.7 %)	2 (5.4 %)	0.471
Urinary tract infection	3 (4.1 %)	0	0.214
Pulmonary embolism	1 (1.4 %)	0	0.478
Superficial incisional SSI	3 (4.1 %)	1 (2.7 %)	0.719
Deep vein thrombosis	0	1 (2.7 %)	0.155
Sepsis	4 (5.4 %)	0	0.150
Septic shock	3 (4.1 %)	4 (10.8 %)	0.167
Unplanned intubation	2 (2.7 %)	3 (8.1 %)	0.196
Transfusion	8 (10.8 %)	12 (32.4 %)	**0.005**
Mortality, n (%)	2 (2.7 %)	4 (10.8 %)	0.075

LOS = length of stay, IQR = interquartile range, INR = international normalized ratio, SSI = surgical site infection; **Bold** indicates statistical significance (p<0.05).

Bold indicates statistical significance (p<0.05).

## Discussion

While the definitive role of NAT in GBC has not been established, it is a proposed strategy for patients with advanced GBC due to potential improvement of resectability and survival. NAT is routinely used for aggressive cancers including gastric, esophageal, and rectal, and has become more prevalent for pancreatic cancer [[7], [8], [9], [10]]. In this propensity matched analysis of four years of national data we found that patients receiving NAT for GBC had similar rates of postoperative bile leakage, liver failure, readmission and mortality compared to a similarly matched group of patients with GBC not receiving NAT. However, we did find that patients receiving NAT had a higher rate and associated risk for requiring postoperative blood transfusion even when controlling for preoperative anemia.

The use of NAT for GBC has been explored in a few prospective studies and many retrospective studies which have shown that NAT may improve tumor resectability and survival outcomes [[12], [13], [14], [15], [16]]. In a prospective feasibility cohort study, Agrawal et al. demonstrated that NAT (chemoradiotherapy with 5-fluorouracil and cisplatinum or chemotherapy alone with cisplatin and gemcitabine) for unresectable GBC resulted in a 15 % resectability rate, with radiologic downstaging seen in over 40 % of tumors with liver involvement and 67 % of those with lymphadenopathy [17]. However, studies addressing postoperative outcomes after resection following NAT for GBC are limited. In a single-institution prospective pilot study, Engineer et al. showed that out of 28 patients with locally advanced GBC treated with neoadjuvant chemoradiotherapy (CRT), 43 % were found to have prolonged postoperative biliary leakage (defined as >5 mg/dL bilirubin in drainage fluid for >7 days) [18]. In contrast, our national analysis found no difference in the rate of bile leakage when compared to a well-matched cohort in terms of demographics and tumor characteristics. In addition, we found no difference in terms of rate of postoperative liver failure, 30-day readmission, or 30-day mortality. However, our study is limited by the low number of complications and mortality, thus preventing any definitive conclusions. The findings appear to warrant a large multicenter trial to better evaluate the effect of NAT on postoperative outcomes in GBC.

The need for postoperative blood transfusion is often multifactorial with considerations based on severity of surgery, surgical technique, tumor location and size, as well as demographic factors such as baseline anemia and/or coagulopathy. However, our analysis did demonstrate an increased rate of blood transfusion in the NAT group when compared to a similarly matched GBC cohort not receiving NAT. This persisted with an over three-fold associated increased risk of transfusion when controlling for anemia. Similar findings regarding increased need for intra/postoperative transfusions after NAT have been seen in a study by Czosynyka et al. regarding pancreatic cancer (NAT 27.4 % vs no NAT 20.3 %, *p* < 0.001) [19]. The reason for this finding was thought to be due to increased tissue firmness and inflammation, which increases friability of tissue and bleeding risk. Alternatively, the difference in transfusion rates could be explained by a difference in severity of surgery between groups. Our study did find a significant difference in rate of major hepatic resection between treatment groups; patients who underwent extensive hepatectomy more often received NAT prior to surgery. Increased transfusion rate in the NAT group could also be related to selection bias as those who received NAT may have had increased concern for vascular involvement. Aggressive solid tumors like GBC rely on neovascularization to grow [20], so these more advanced tumors could have a greater degree of angiogenesis and therefore bleeding risk. Finally, increased risk for transfusion may be associated with the properties of chemotherapy treatments used for GBC including gemcitabine and cisplatin, which are known suppressors of bone marrow [21,22]. Future studies are needed to confirm this finding.

There are many limitations to our study including those inherent to a large retrospective database, such as selection bias, missing data, and miscoding. Another significant limitation is the small sample size and limited power, which could lead to a Type II error of failing to detect small differences in mortality and complications between the treatment groups. The small sample size is a result of the rarity of GBC within the United States and the even more rare use of NAT. In addition, the database involves a heterogeneous population receiving different therapy protocols and groups patients who received chemotherapy with those who received local ablation therapies. Also, the database is missing pertinent variables including indications for NAT, treatment type including details regarding the chemotherapy agent used, duration, and dose, as well as complications related to NAT. This analysis was also limited by a relatively short time frame of data collection from 2014 to 2017. An additional major limitation is that the database also does not include patients who received NAT but ultimately did not undergo surgery, thus not accounting for cases where NAT acted as a selection process for tumor biology characteristics and/or led to significant complications. The database is also limited to 30-day outcomes and thus does not provide any long-term functional, cancer free survival or overall survival outcomes. Finally, the small sample size does subject this research to the potential for a Type II error.

## Conclusions

In a propensity matched cohort analysis of patients undergoing surgical resection for GBC, patients receiving NAT had similar rates of postoperative bile leakage, liver failure, 30-day readmission, and mortality when matched to a similar cohort of patients undergoing surgery without receiving NAT. Although, patients receiving NAT had a higher rate and associated risk for postoperative blood transfusion. However, this study appears underpowered to draw definitive conclusions and thus future large prospective studies are needed to better evaluate the effects of NAT on perioperative outcomes for GBC patients.

## Funding

This research did not receive any specific grant from funding agencies in the public, commercial, or not-for-profit sectors.

## Ethics approval

This study was deemed exempt from full review by the Institutional Review Board at the University of California Irvine.

## CRediT author contributions

All co-authors contributed to the conceptualization, methodology, data curation, formal analysis, critical review and editing with final approval of the manuscript. NY, AG, and JN contributed to the writing of the original manuscript. AG and JN contributed to the supervision of this project.

## Declaration of competing interest

We have no conflicts of interest to disclose.

## Data Availability

The data that support the findings of this study are available from the American College of Surgeons. Restrictions apply to the availability of these data, which were used under license for this study. Data are available https://www.facs.org/quality-programs/acs-nsqip/participant-use with the permission of The American College of Surgeons.
